# Description of a new species of *Petrolisthes* in the Indo-West Pacific with a redefinition of *P. hastatus* Stimpson, 1858 and resurrection of *P. inermis* (Heller, 1862) (Crustacea, Anomura, Porcellanidae)

**DOI:** 10.3897/zookeys.516.9923

**Published:** 2015-08-10

**Authors:** Bernd Werding, Alexandra Hiller

**Affiliations:** 1 Institut für Tierökologie und Spezielle Zoologie der Justus-Liebig-Universität Giessen, Heinrich-Buff-Ring 29 (Tierhaus), D-35392 Giessen, Germany; 2 Smithsonian Tropical Research Institute, Apartado 0843-03092, Panama, Republic of Panama

**Keywords:** Crustacea, Decapoda, Porcellanidae, new species, Indo-West Pacific

## Abstract

The porcellanid crab *Petrolisthes hastatus* Stimpson, 1858, has been traditionally viewed as a highly variable species with a wide distribution in the West Pacific. For more than a century there has been taxonomic confusion of this species with morphologically similar taxa, some of which were synonymized with Stimpson’s taxon. We redefine *Petrolisthes hastatus*, resurrect *Petrolisthes inermis* as a valid species, discuss the status of *Petrolisthes tenkatei* De Man, 1893, and describe a new species as *Petrolisthes elegantissimus* from Indonesia.

## Introduction

Porcellanidae is a morphologically and ecologically diverse family of decapod Crustacea containing approximately 280 species in 23 genera with littoral or sublittoral distributions throughout the tropical and temperate regions of all oceans (e.g. Haig 1956, 1960; Werding 1977; Werding et al. 2003; Osawa and Chan 2010; Osawa and McLaughlin 2010). This family comprises one of the most abundant groups of crustaceans in rocky and coral habitats. The genus *Petrolisthes* Stimpson, 1858, is the most species-rich in the family (> 100 species), contains several complexes of species that are morphologically highly variable and widely distributed, and has a long and complicated taxonomic history (e.g. Haig 1960; Stillman 2001; Hiller et al. 2006).

For more than a century, *Petrolisthes hastatus* Stimpson, 1858 has been object of taxonomic confusion. This species has been known to have large intraspecific variation. In his original description Stimpson briefly characterized the species, and in a later paper (Stimpson 1907) this description was essentially repeated with the addition of a figure (Pl. XXII, fig. 4) that exhibits the main characteristics of the species including a flattened, spineless carapace with a triangular rostrum, large, flattened chelipeds, each with the carpus armed with three evenly distributed teeth at the anterior margin, and with a single, prominent curved distal spine at the posterior end. The presence of pubescence in the gape of the cheliped fingers was not mentioned by Stimpson (1858, 1907), and the walking legs were characterized as “hairy, sparsely spinulose above”.

Subsequent to Stimpson’s original description, Heller (1862) described *Petrolisthes inermis*, a species Heller characterized as close to *Petrolisthes rufescens* (Heller, 1861), but with three instead of four teeth at the anterior margin of the cheliped carpus (the two species were originally referred to the genus *Porcellana*). De Man (1888) compared six specimens from the Nicobar Islands with a type of *Petrolisthes inermis*, and considered the species to be potentially synonymous with *Petrolisthes hastatus*. De Man (1893) described *Petrolisthes tenkatei* from the Malay Archipelago and discussed the possibility of his new species being a synonym of *Petrolisthes hastatus*.

Succeeding studies (Haig 1964, 1979; Miyake 1943) considered *Petrolisthes inermis* a synonym of *Petrolisthes hastatus*. Nakasone and Miyake (1971) formally synonymised *Petrolisthes tenkatei* with *Petrolisthes hastatus*. This concept of synonymies has been retained in the recent list of porcellanid species of the world provided by Osawa and McLaughlin (2010).

Most figures of *Petrolisthes hastatus*, e.g. Miyake (1943, fig. 5), Haig (1979, fig. 5), Hsieh et al. (1998, fig. 18), and Osawa and Chan (2010, figs 98–102), exhibit a phenotype that largely agrees with Stimpson’s (1907) figure (Pl. XXII, fig. 4), while the specimens photographed by Nakasone and Miyake (1971, pl. 1 C, D and also probably B) seem to represent another phenotype, with each cheliped having a slender carpus (more than three times longer than broad) armed with two low teeth on the anterior margin, and an elongated chelae, each with a large tuft of setae in the gape of the fingers, visible from dorsal side.

While examining collections of Porcellanidae from the Naturalis Biodiversity Center (Leiden) and the Muséum National d’Histoire Naturelle (Paris), we found three morphotypes from Indonesia and Papua New Guinea that fit the general characters of *Petrolisthes hastatus* as described in the literature. However, a detailed examination of these morphotypes revealed that they correspond to three distinct species: *Petrolisthes hastatus* Stimpson, *Petrolisthes inermis* (Heller) and a third new species described herein as *Petrolisthes elegantissimus*. These three species share the following characters: carapace without spines; carpus of cheliped with widely-set, low teeth on the anterior margin, the number of teeth varying, the posterior margin slightly curved outside, and with a single, prominent distal tooth; merus of walking legs unarmed or with a varying number of spines on dorsal margin, and with a distal spine on the ventral margin, at least in first legs.

Type material of *Petrolisthes hastatus* was searched for in the collections of the Smithsonian National Museum of Natural History, but it seems to be inexistent (R. Lemaitre, per. comm.). In the collections of the Naturalis Biodiversity Center (Leiden) there are series of types corresponding to the original series collected by H. ten Kate in 1891, on which the description of *Petrolisthes tenkatei* by De Man (1893) was based, and which is clearly distinct from *Petrolisthes hastatus* as defined here. Regarding *Petrolisthes inermis* (Heller, 1862), there are three specimens from the Novara Expedition from the Nicobars (Naturhistorisches Museum, Vienna, Austria), which were examined.

## Material and methods

Most material examined is deposited in the Naturalis Biodiversity Center (NBC) in Leiden, the Netherlands, and the Muséum National d´Histoire Naturelle (MNHN) in Paris, France. This material was compared to old samples from the Naturhistorisches Museum (NHM) in Vienna, Austria, which corresponds to the original material collected by the Novara Expedition, and labelled as syntypes of *Porcellana inermis* Heller, 1862 by later curators. Additionally, other NBC specimens collected by H. ten Kate, and presently designated as lectotype and paralectotypes of *Petrolisthes tenkatei* De Man, 1893, were also examined. Two paratypes of the new species, were deposited in the collections of the Senckenberg Naturmuseum (SNM), Frankfurt, Germany. In the synonymy we included only those citations in which are could confirm that the respective species were treated in former reports.

Measurements are given as carapace length (CL) × carapace width (CW) for representative and/or largest specimens of each species. Ovigerous females are denoted as “ov”, and the three pairs of walking legs as L1-L3.

## Data resources

The data underpinning the analysis reported in this paper are deposited in the Dryad Data Repository at http://dx.doi.org/10.5061/dryad.k71m0

## Results

### Systematic account

#### Petrolisthes hastatus

Taxon classificationAnimaliaDecapodaPorcellanidae

Stimpson, 1858

Fig. 1

Petrolisthes hastatus Stimpson, 1858: 228, 241; 1907: 184, pl. 22 fig. 4. – Miyake 1943: 54, 62, figs 5, 6. – Haig 1964: 360 (partim); 1979: 124, fig. 5. – Johnson 1970: 13. – Nakasone and Miyake 1971: 5 (partim), pl. 1, fig. A. – Hsieh, Chan and Yu 1998: 307, figs 14D, 18A–H. – Osawa and Chan 2010: 131, figs 98–102.

##### Material examined.

**Indonesia: Snellius Expedition 1929–1930.** RMNH.CRUS.D.56378, ca. 260 specimens, Sissie by Misool, beach, 06. Oct. 1929; RMNH.CRUS.D.56379, ca. 140 specimens; RMNH.CRUS.D.56380, ca. 60 specimens, Ambon, 11.-17.09.1930; RMNH.CRUS.D.56381, ca. 145 specimens; RMNH.CRUS.D.56382, ca. 90 specimens. Aloonf, beach and reef, 08.02.1930; RMNH.CRUS.D.56383, ca. 90 specimens, Tidore, strand, 24.–29.09.1929; RMNH.CRUS.D.56384, 9 males, 9 females (3ov), Pelee (by Misool), beach, 04.10.1929; RMNH.CRUS.D.56386, 1 male, Menado, 10.10.1930; RMNH.CRUS.D.56390, 1 male, Morotai, 03.-10.06.1930 RMNH.CRUS.D.56396, 2 spec. Bopyridae, Alsang, beach and reef, 08.02.1930; RMNH.CRUS.D.56397, 1 male, 1 female (ov), Los (by Misool), beach and reef, 03.-06.10.1929; RMNH.CRUS.D.56398, 4 males with Bopyridae, Tidore, beach, 24.-29.09.1929; RMNH.CRUS.D.56399, 1 male with Bopyridae, Paleleh, Celebes, beach, 21.0.1929; RMNH.CRUS.D.56400, 1 male, Maenado, 01. Oct. 1930; RMNH.CRUS.D.56401, 1 male, Ambon, 11.–17.09.1930; RMNH.CRUS.D.56402, 2 males with Bopyridae, Pelokan, Postiljon Island, beach and reef, 20.12.1929; RMNH.CRUS.D.56403, 1 male, near Koepang, strand, 25.11.1929; RMNH.CRUS.D.56404, 1 male, Tidore near Koepang, Tjabo, beach, 24.- 29.09.1929; RMNH.CRUS.D.56405, 1 male, Paleleh Celebes, beach, 21.08.1929; MNH.CRUS.D.56406, 1 male, Ake Salaka, Raoebaai Halmakeira, beach and reef, 28.05.1930; RMNH.CRUS.D.56407, 1 male, Taliaboe, Pasik Lpah,Solea Island), beach, 19.03.1930. **Papua New Guinea.** MNHN-IU-2013-9128, 1 male, Stn. PM08, 05°15'17.82" - 145°46'38.91E’’, Yabob Village, Gum River, 0–1m, 12.11.2012; MNHN-IU-2013-960, 1 male; MNHN-IU-2013-9539, 1 female (ov), Stn. PM41, 05°08.1'S - 145°49.3'E, Wonad Island, sandy beach and intertidal rocks, 0-1m, 27.11.-09.12.2012; MNHN-IU-2013-295, 1 male, Stn. PM12 05°00.2'S - 145°47.6'E, Rempi Area, S Dumduman Island, limestone rocky intertidal, 0-1m, 09.11.2012; MNHN-IU-2013-9615, 1 male, Stn. PM22, 05°04.7'S - 145°48.9'E Sek I, Night Tide, 14.11.2012. MNHN-IU-2013-9615, 1 male, Stn. PM22, 05°04.7'S - 145°48.9'E Sek I, Night Tide, 14.11.2012; MNHN-IU-11212, 1 male; Stn. VM46, 15°34'S- 167°12'E, Vanuatu, Aoré Island, 03.10.2006.

##### Measurements.

Largest male: CL 10.7 mm × CW 10.9 mm; largest female: CL 10.3 mm × CW 10.7 mm.

##### Description.

Carapace as broad as long or slightly broader than long, evenly rounded on branchial regions, broadest at posterior branchial level; surface covered with flattened, fine granules and faint plications. Front strongly produced, sinuously triangular, rostrum with a median sulcus, supraocular angle scarcely produced, depressed by a shallow groove. Orbits shallow; outer orbital angle rounded, scarcely produced, forming a low lobe with continuing hepatic margin; epibranchial angle accentuated but without notch or spine, continuing in a ridge along mesobranchial margin; branchial margin unarmed. Protogastric ridge forming a distinct crest, cervical grooves and regions slightly defined. Lateral walls with short, feathered setae.

Basal segment of antennular peduncle with faint transverse rugae; anterior margin rounded, with a distinct tooth at mesial corner and a rounded protuberance at lateral corner.

First movable segment of antenna with foliate, subquadrate projection without prominent tooth; second segment with a longitudinal granular crest ending proximally in a rounded tooth; third rounded, unarmed.

Chelipeds sub-equal. Merus with transverse, low granules on dorsal surface, anterior margin armed distally with a prominent, finger-shaped, rounded lobe; dorso-distal margin fringed with short setae. Carpus about 2.5 to 3 times longer than broad; dorsal surface covered with shallow, transverse rows of granules; anterior margin with 3 (rarely 4) wide-set, serrate-edged, hooked teeth, the proximal one normally the largest; posterior margin slightly curved outwards, granules along posterior margin enlarged, forming a crest terminating in a prominent, curved tooth; dorso-distal margin with short pubescence posteriorly.

Chelae large, broad and flattened; outer margin evenly arcuate and unarmed; dorsal surface covered with shallow, rounded granules; fingers broad, spineless, meeting at their entire length or slightly gaping in the larger chela, entire gape covered with a short pubescence.

Ischium of walking legs covered with feathered setae; merus spineless or with 1-3 irregularly-set spines and a fringe of feathered setae along the anterior margin; merus of L1 and L2 with a posterodistal spine. Carpus with a fringe of feathered setae on the anterior margin. Propodus and dactylus with scattered, feathered and long, simple setae; propodus ventrally with a distal triplet of movable spinules, and one additional spine at mid-distance; dactylus with 3 movable spines on posterior margin.

**Figure 1.**
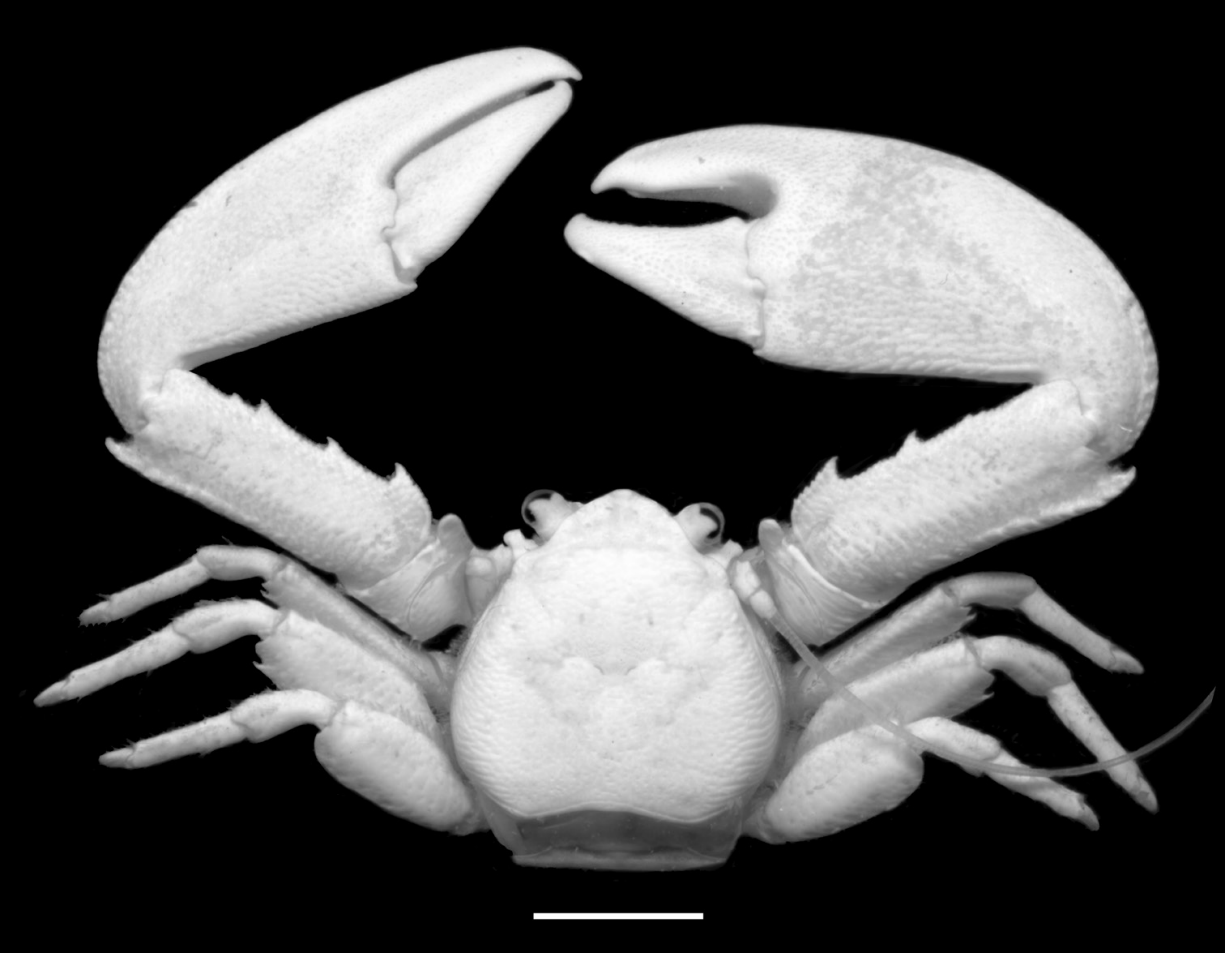
*Petrolisthes hastatus* Stimpson, 1858. Dorsal view of male RMNH.CRUS.D.56380, Ambon, Indonesia. Scale bar - 5 mm.

##### Variation.

The number of teeth on the anterior margin of the cheliped carpus may be reduced, the position of the lacking tooth is then marked by a small knob; in other cases a vestigial additional tooth is present.

##### Habitat.

*Petrolisthes hastatus* is a shallow water species. The specimens of the Snellius and Papua New Guinea expeditions were collected from intertidal or shallow subtidal (0.5 m depth) rocks and reefs. Haig (1979) and Osawa and Chan (2010) described the habitat of the species as intertidal, under rocks.

##### Distribution.

The species, as defined here, is restricted to the western Pacific, from Singapore, eastwards trough Indonesia and Papua New Guinea to Vanuatu. Northwards it occurs in Taiwan, and Ryukyu and Kikaijima, Japan.

#### Petrolisthes inermis

Taxon classificationAnimaliaDecapodaPorcellanidae

(Heller, 1862)

Fig. 2

Porcellana inermis Heller, 1862: 424 (partim); 1865: 76, pl. 6, fig. 5.
Petrolisthes inermis ? De Man, 1893: 288, pl. 7, fig.1.
Petrolisthes tenkatei De Man, 1893: 289, pl. 7, figs 2, 2a, 2b.
Petrolisthes sp. n.? De Man, 1902: 69, pl. 23, fig. 37.
Petrolisthes hastatus Nakasone & Miyake, 1971: 5 (partim), pl. 1, B-D.

##### Type material.

**Nicobar Islands: Novara Expedition 1857–59.** NHM24237, lectotype, female (ov), CL 6.4 mm × CW 7.0 mm, 23.02.–20.03.1858; NHM24238, paralectotype, 1 male, CL 6.7 mm × CW 6.6 mm, 23.02.–20.03.1858.

##### Other material examined.

**Indonesia.** RMNH.CRUS.D.2604, 1 male, Indonesia, Endeh, Flores Island, leg. H. ten Kate, 1891; Measurements: CL 8,5 mm × CW 9,0 mm; RMNH.CRUS.D.1643, 5 males, 2 females (1ov), Indonesia, Endeh, Flores Island, leg. H. ten Kate, 1891; RMNH.CRUS.D.56410, 4 males, Indonesia, Sangihe Island, leg. D.J. Hoedt 1867. Indonesia: Snellius Expedition. RMNH.CRUS.D.56411, 12 males, 3 females (ov), Ternate, beach, 24.09.1929; RMNH.CRUS.D.56412, 1 male, Rambay by Timor, beach and reef, 26-28.11.1929; RMNH.CRUS.D.56413, 1 male, 2 females (ov), Paleleh, Celebes (Sulawesi), beach, 22.08.1929; RMNH.CRUS.D.56414, 1 male, 1 female, (with Bopyridae), Ende, (Flores), 06.-08.11.1930; RMNH.CRUS.D.56415, ca. 430 specimens, Ende, (Flores), 06.-08.11.1930; RMNH.CRUS.D.56416, 2 males, Ambon, beach and reef 0-2m, 06.05.1930; RMNH.CRUS.D.56417, ca. 80 specimens, Ambon, 11.-17. 09. 1930. **Indonesia: Indonesien-Dutch Snellius-II Expedition.** RMNH.CRUS.D.56418, 3 males, Sta.4.001, Ambon Bay, near Tawiri, cobble beach to disturbed reef, dead corals, 0-5m, snorkeling, scuba diving, 22. and 30.08.1984. RMNH.CRUS.D.56419, 1 male, Sta.18, Ambon, Hitu, E side of Laha, up to and including Tawiri, littoral, 08.11.1990; RMNH.CRUS.D.56420, 12 males, 6 females (5ov), Ambon, Hitu, W side of Laha, 06.12.1990; RMNH.CRUS.D.56421, 2 males (1 without chelae), 1 female (ov), Ambon, Sta. 36, Paso,(Bugala), littoral collection, 05.12.1990; RMNH.CRUS.D.56422, 1 male, Ambon, Station 1, in front of the house, littoral collection, leg. CHJM Fransen, 06.11.1990. **Papua New Guinea.** MNHN-IU-2013-9125, 3 males, 1 female (ov), Stn. PM08, 05°15'17.82" – 145°46'38.91", Yabob Village, Gum River, 0-1m, 12.11.2012.

##### Measurements.

Largest male: CL 11.0 mm × CW 11.0 mm; largest female: CL 8.6 mm × CW 9.0 mm.

##### Description.

Carapace as broad as long, or somewhat broader than long, evenly rounded at branchial regions, broadest at posterior branchial level; surface covered with faint plications, more accentuated laterally. Front produced, sinuously triangular, rostrum with a moderately deep median sulcus, supraocular angle scarcely produced, depressed by a shallow groove. Orbits shallow, outer orbital edge bluntly produced, forming a shallow lobe with hepatic margin. Epibranchial angle unarmed, marked by a ridge continuing along the mesobranchial margin. Protogastric ridge forming a distinct crest, cervical grooves and regions poorly defined. Lateral walls thickly matted with long, feathered setae, largely concealing the basal parts of the walking legs.

Basal segment of antennular peduncles with faint transverse rugae, anterior margin rounded, with distinct tooth at mesial corner and rounded protuberance at lateral corner.

First movable segment of antenna anteriorly with foliate, square-cut projection with a shallow, forwardly directed tooth; second with a longitudinal granular crest, extending proximally in a rounded tooth, third rounded, unarmed.

Chelipeds sub-equal. Merus with transverse, shallow plications on dorsal surface, anterior margin armed distally with a finger-shaped, granular lobe, fringed with short setae. Carpus slender, highly variable, from about 3 to 4 times longer than broad, dorsal surface covered with low, scale-like granules; anterior margin with 2 shallow teeth, a third one faintly marked or lacking; the proximal tooth normally the largest and acute, the second one smaller and blunt. Posterior margin slightly curved outwards, granules along posterior margin enlarged, forming a crest along the postero-distal margin, extending into a spine-tipped, distal tooth. Chelae large, slender, transversely swollen; outer margin curved on entire length, unarmed; fingers spineless, frequently gaping in larger chela. Gape of fingers with large, dense pubescence, visible from above, sometimes only in one chela, seldom lacking. Ischium of walking legs covered with a pubescence of feathered setae; merus with a single dorsal spine close to the distal edge in L1 and L2, and a fringe of feathered setae on anterior margin; merus of L1 and L2 with a posterodistal spine, sometimes lacking in L2 or in both. Carpus and propodus with a fringe of feathered setae on anterior margin, with scattered feathered and simple setae. Propodus ventrally with distal triplet of movable spinules and one additional spine at mid-distance; dactylus with 3 movable spines on posterior margin.

**Figure 2.**
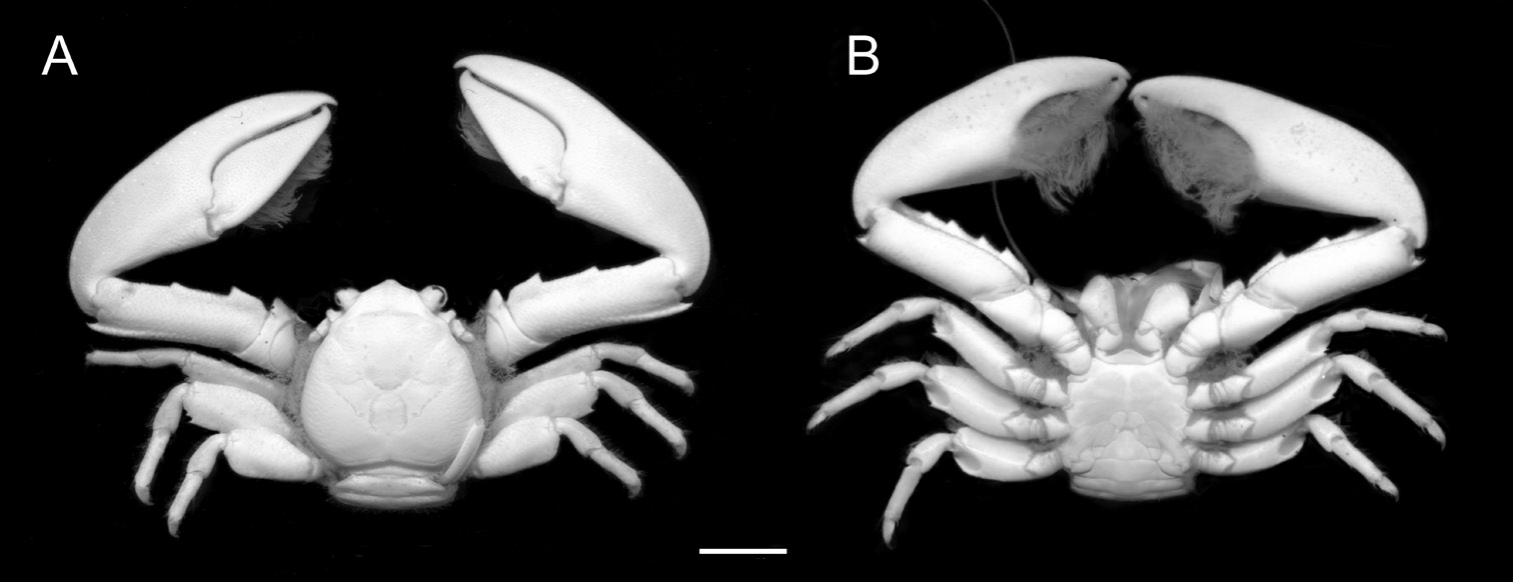
*Petrolisthes inermis* (Heller, 1862). Dorsal (**A**) and ventral (**B**) views of female RMNH.CRUS.D.56417, Ambon, Indonesia. Scale bar - 5 mm.

##### Variation.

Large specimens normally present more elongate and narrower chelipeds, more variation in the form of the chelipeds than smaller ones, and often exhibit a remarkable heterochely.

##### Habitat.

*Petrolisthes inermis* seems to be a shallow water species. According to the material of the Papua New Guinea Expedition and the two Snellius Expeditions in Indonesia the species was collected in the littoral in rocky beaches and on dead corals, in depths of 0–5 m.

##### Distribution.

Eastern Indian Ocean, Nicobar Islands, Indonesia, Papua New Guinea, New Caledonia.

##### Remarks.

Some of the original material from the Novara Expedition is deposited in the collections of the Naturhistorisches Museum (NHM) in Vienna. We found two lots labelled as “Syntypus” of *Porcellana inermis* Heller, 1862. One lot (NHM24237) contained a moderate-sized female, which was selected as lectotype. The other (NHM24238) contained a small male lacking the right chela and two walking legs, and a larger female lacking one cheliped. The small male was selected as paralectotype. The second specimen represents a different species. De Man´s (1893) original specimens of *Petrolisthes tenkatei* (RMNH.CRUS.D.2604, RMNH.CRUS.D.1643) are deposited in the collections of the Naturalis Biodiversity Center (Leiden). They are labelled as lectotype and paralectotypes of *Petrolisthes tenkatei*, which is a synonym of *Petrolisthes inermis*. Judging from the figure of *Petrolisthes* sp. n. from De Man (1902, fig. 37) this species also corresponds to *Petrolisthes inermis*, and is therefore included in the synonymy of that species.

#### Petrolisthes elegantissimus sp. n.

Taxon classificationAnimaliaDecapodaPorcellanidae

http://zoobank.org/3EAE708F-6E86-4545-9CE7-43939FBEB380

Figs 3 and 4

##### Type material.

**Indonesia.** RMNH.CRUS.D.56374, holotype, male, CL 8.9 mm × CW 8.7 mm, NW Guinea, Pulau Japen, coast near Sarawandori, leg. W. van Seroei, 24.2.1955. Paratypes: RMNH.CRUS.D.56408, 4 males, 5 females (3 ov), same data as holotype. RMNH.CRUS.D.56375, 2 males, CL 8.8 mm × CW 8.8 mm, and CL 6.3 mm × CW 6.0 mm, same data as holotype. **Indonesia: Snellius Expedition.** RMNH.CRUS.D.56376, 5 males, 3 females (ov), Los (near Misool), 3–6.10.1929; RMNH.CRUS.D.56377, 2 males, Wotap, (Pulau Wotap), Tanimbar Island, beach and reef, 20.–23.10.1929; RMNH.CRUS.D.56409, 3 males, 1 female (ov), Pelee near Misool, beach, 04.10.1929. SMF 48329, 1 male, 1 female (ov), CL 8.1 mm × CW 8.6 mm, Sissie near Misool, beach, 06.10.1929.

##### Measurements.

Largest male: CL 8.9 mm × CW 8.7 mm; largest female (ov): CL 8.1 mm × CW 8.6 mm.

##### Description.

Carapace as long as broad or slightly broader than long, invertedly heart-shaped, broadest at metabranchial level; dorsal surface granular, branchial regions with low striae on outer margin. Front strongly produced, sinuously trilobate; lateral lobes formed by the supra-ocular edge; rostrum dorsally with deep median sulcus extending beyond protogastric ridge; orbits shallow, nearly straight, outer orbital angle rounded, forming a shallow lobe extending to hepatic margin. Epibranchial angle distinct but without a notch or spine, continuing in a ridge along the branchial margin. Protogastric ridge, cervical grooves and regions well marked. Lateral walls with scattered, simple setae.

Telson (Fig. 4C) composed of 7 plates; lateral plates narrow; lateral margins of central plate emarginate.

Basal segment of antennular peduncle (Fig. 4B) with faint transverse rugae; anterior margin rounded, granular, with minute teeth at mesial and lateral corners.

First movable segment of antenna with a lamellar, spine-tipped lobe, second with a longitudinal granular crest extending proximally into a rounded tooth, third rounded, unarmed.

Chelipeds sub-equal, merus with transverse, low granules on dorsal surface; anterior margin armed distally with a prominent, spine-tipped lobe. Carpus straight, margins subparallel, about 4–5 times as long as broad; dorsal surface covered with small, verruciform granules; anterior margin armed with 3–5 irregularly-set, acute small teeth of similar size; posterior margin slightly curved outwards with larger granules forming a crest along the distal half of length extending in a prominent, curved, distal tooth. Chela large, slender, posterior margin weakly curved, unarmed; dorsal surface covered with low, spherical granules, with a low, median crest extending to the base of the dactylus; fingers unarmed, gape without or with very short pubescence.

Walking legs extremely long and slender. Ischium devoid of setae or with few scattered, plumose setae. Merus devoid of setae or with few simple setae, unarmed or with a varying number (1–4) of irregularly-set, sharp spines along anterior margin with a prominent postero-distal spine in L1, weakly developed or lacking in L2, and postero-distally rounded in L3. Carpus, propodus and dactylus with scattered, simple setae. Propodus with distal triplet of movable spinules, and one additional spine on median part of posterior margin. Dactylus with 3 movable spines on posterior margin.

**Figure 3.**
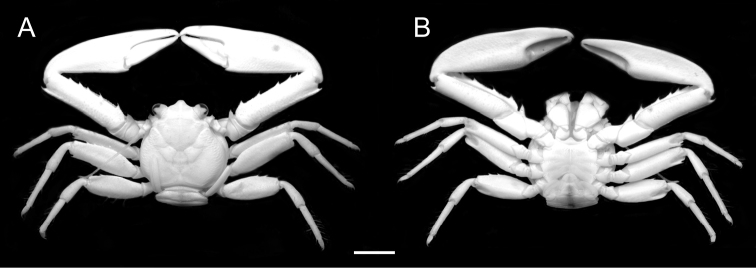
*Petrolisthes elegantissimus* sp. n. Dorsal (**A**) and ventral (**B**) views of paratype male RMNH.CRUS.D.56375, NW Guinea, Indonesia. Scale bar - 4 mm.

**Figure 4.**
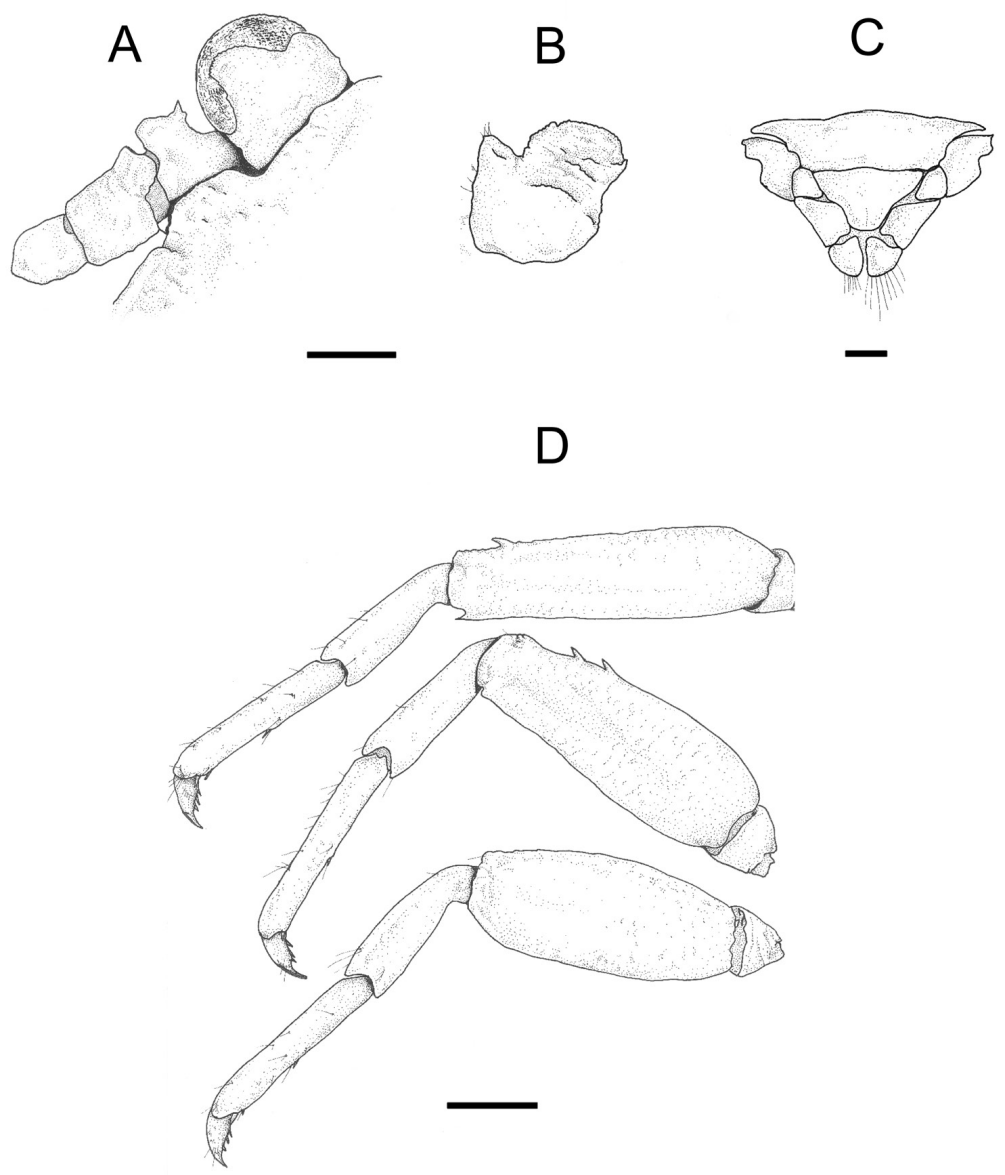
*Petrolisthes elegantissimus* sp. n., paratype male RMNH.CRUS.D.56408, NW Guinea, Indonesia. **A** Dorsal view of left ocular peduncle and basal segments of left antennal peduncle **B** Dorsal view of basal segment of left antennular peduncle **C** External view of sixth abdominal segment with telson **D** Lateral view of first to third left walking legs. Scale bar - 1 mm (**A–C**), 2 mm (**D**).

##### Variation.

Large specimens normally present more elongate and narrower chelipeds than smaller ones. The teeth of the anterior margin of the cheliped carpus often varies in the same specimen in number and position.

##### Habitat.

*Petrolisthes elegantissimus* sp. n., like the other two species, seems to be a shallow water species.

##### Distribution.

Only known from a restricted region in eastern Indonesia.

##### Etymology.

The specific name is derived from the Latin *elegans* (tasteful, refined), referring to a more elegant and gracile general habitus compared with that of the related species, *Petrolisthes hastatus* and *Petrolisthes inermis*. The species name is an adjective in the nominative singular.

## Remarks

The status of *Petrolisthes hastatus*, *Petrolisthes inermis* and *Petrolisthes tenkatei* has been unclear for more than a century. The comparison of old and new specimens with part of the original material from Heller in the NHM, Vienna, and from De Man from NBC, Leiden, revealed that *Petrolisthes inermis* is a valid species, and that *Petrolisthes tenkatei* falls into synonymy with the latter. The main characters that allow distinguishing *Petrolisthes hastatus*, *Petrolisthes inermis* and *Petrolisthes elegantissimus* sp. n. are: 1) Anterodistal lobe of the merus of the cheliped: in *Petrolisthes hastatus* and *Petrolisthes inermis* it is finger-shaped and rounded; in *Petrolisthes elegantissimus* sp. n. it is subtriangular and spine-tipped. 2) Cheliped carpus: in *Petrolisthes hastatus* it is approximately 2.5–3 times longer than broad, the anterior margin bearing three, wide-set, serrate-edged, hooked teeth with the proximal one normally being the largest; in *Petrolisthes inermis* the carpus is slender, nearly 3–4 times longer than broad, armed on anterior margin with two shallow teeth, the proximal one being the largest and forming an acute tooth, the second one being smaller and blunt; in *Petrolisthes elegantissimus* sp. n. it is slender, 4–5 times longer than broad, armed on the anterior margin with 3–5, irregularly-set, acute small teeth of similar size. 3) Chela: in *Petrolisthes hastatus* it is broad, flattened, the gape of the fingers being covered with a short pubescence; in *Petrolisthes inermis* it is slender, transversely swollen, with the gape of fingers with large, dense pubescence, normally visible from above; in *Petrolisthes elegantissimus* sp. n. it is large, slender, with the gape without or with short pubescence. 4) Merus of walking legs: in *Petrolisthes hastatus* it is spineless or with 1-3 irregularly-set, small spines, merus of L1 and L2 bearing a posterodistal spine; in *Petrolisthes inermis* it bears a single dorsal spine close to the distal edge in L1 and L2, and merus of L1 and L2 bears a posterodistal spine; in *Petrolisthes elegantissimus* sp. n. it is long, slender, with anterior margin spineless or with a varying number (1–4) of irregularly-set, prominent spines; in L1 it bears a prominent posterodistal spine, in L2 it is weakly developed or lacking, and in L3 it is posterodistally rounded.

## Discussion

The geographic range of the three species here treated suggests that they are sympatric in Indonesian waters. *Petrolisthes hastatus* has the widest range in the West Pacific while *Petrolisthes inermis* and *Petrolisthes elegantissimus* sp. n. seem to have a more limited distribution. *Petrolisthes inermis* is the only one of the three species extending its range to the eastern Indian Ocean. Ecologically, the three species seem to prefer shallow-water habitats characterized by reefs, and rocks on sand. New sampling efforts that document coloration and compile more precise ecological information, combined with molecular analyses that corroborate species monophyly, will further aid in clarifying the taxonomic status, ecological preferences and geographic boundaries of the three species, and in proposing possible speciation scenarios.

## Supplementary Material

XML Treatment for Petrolisthes hastatus

XML Treatment for Petrolisthes inermis

XML Treatment for Petrolisthes elegantissimus
